# Modeling human placental biology: a review of organoid technologies

**DOI:** 10.3389/fcell.2025.1693923

**Published:** 2026-01-05

**Authors:** Alaijah Bashi, Tyana Joseph, Viviane Schuch, Erica L. Johnson

**Affiliations:** Department of Microbiology, Biochemistry and Immunology Morehouse School of Medicine, Atlanta, GA, United States

**Keywords:** organoids, trophoblast models, maternal–fetal interface, placenta, stem cell

## Abstract

The development of placental organoids represents a significant breakthrough in reproductive biology, offering an advanced platform for studying human placental development and function. Unlike traditional two-dimensional (2D) cell cultures, three-dimensional (3D) organoid models, such as primary trophoblast organoids (TOs), JEG-3 organoids, and stem cell-derived organoids, provide a more robust and physiologically relevant environment. These models enable researchers to mimic the human placenta’s complex architecture and cellular interactions. As a result, these advanced models promise to reveal the mechanisms underlying placental development and the associated disorders. This review compares different organoid types, highlighting their unique features and applications in studying trophoblast invasion, syncytialization, and placental barrier function. Bioinformatics approaches, particularly transcriptomic analyses, have been crucial in validating these models and identifying stage-specific markers of placental development. With challenges such as standardization issues and ethical considerations persisting, the integration of multiple organoid models, advanced technologies, and computational analyses currently provides the most comprehensive strategy for mimicking placental development across all stages *in-vivo*. Future directions for organoid technologies include the development of multi-organ-on-a-chip models and personalized medicine applications. This review concludes that while no single method perfectly replicates all stages of placental development, the combination of various 3D organoid models, supported by advanced technologies and computational analyses, offers the most effective approach to studying placental biology *in-vivo*.

## Introduction

Organoids have become a groundbreaking advancement in biomedical research, enabling the study of complex biological processes in a controlled *in-vivo* environment. These 3D self-organizing cell structures resemble miniature organs, replicating essential structural and functional features of their *in-vivo* counterparts (Tang et al., 2022). Among these organoid models, placental organoids have emerged as powerful tools for mimicking key aspects of human placental development and function. Notable characteristics include the presence of chorionic villi and the secretion of crucial pregnancy hormones like human chorionic gonadotropin (hCG) (Huang et al., 2023). Inspired by the distinct properties of primary placental cells, researchers have developed sophisticated *in-vivo* models, such as organoid systems, to more accurately reflect the intricate nature of the placenta and overcome the limitations of conventional 2D cultures and animal models. This innovation marks a pivotal step forward in cell biology and tissue engineering, bridging the gap between simplified cell cultures and complex living organisms (Tang et al., 2022; Arjmand et al., 2023). This advancement in organoid technology builds on decades of research, with its roots tracing back to foundational cell culture studies in the mid-20th century.

**FIGURE 1:**
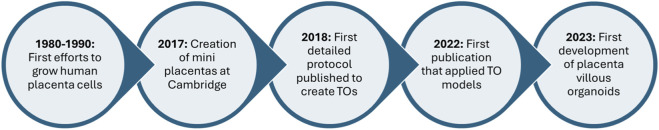
A timeline of the evolution of placenta organoid research. The first preliminary research began in the late 1980s and has sharply increased since 2017, with current research focusing on the application of placenta organoids.

The concept of organoids dates back to the mid-20th century, originating from early tissue culture experiments in which scientists observed cells self-organizing into basic structures resembling tissues or organs when cultured *in-vivo* (Millet and Gillette, 2012; Witkowski, 1979). However, it was not until the late 2000s that groundbreaking studies demonstrated the ability of stem cells to form complex, self-organizing structures (Simian and Bissell, 2017). Notably, researchers showed that human induced pluripotent stem cells (iPSCs) could differentiate into neural cells and organize into polarized cortical tissues (Mariani and Vaccarino, 2019). More recently, a 2023 study revealed that human pluripotent stem cells could self-organize into 3D structures mimicking early post-implantation embryonic development (Pedroza et al., 2023). Since then, organoid technology has advanced rapidly, with protocols now available for generating miniature models of various organs—including the brain, liver, kidney, and, more recently, the placenta (Tang et al., 2022; Yang et al., 2024). This timeline illustrates the remarkable advancements in organoid research over the past century (Figure 1). These developments have been especially impactful in areas such as placenta research, where there is a critical need for consistent and accurate models to investigate the maternal-fetal interface.

In placenta research, it is essential to understand placental function, uncover the root causes of pregnancy complications, and explore maternal-fetal crosstalk (O’Brien and Wang, 2023). This requires a model that is both reproducible and accessible for studying the maternal-fetal interface (Dusza et al., 2023). The placenta is a complex, dynamic organ vital to fetal development, supporting gas exchange, providing immune protection, and serving as a physical barrier between mother and fetus (Gude et al., 2004). However, accurately modeling the placenta remains challenging due to its unique structure and multifaceted functions (Gallagher et al., 2023). These difficulties stem from the limitations of animal models, which cannot fully replicate human placental architecture, and from restricted access to primary human placental tissue (Aye, 2024; Costa et al., 2021). In addition to these challenges, conventional *in-vivo* approaches also fall short in replicating the complexity of the human placenta.

Traditional 2D cell cultures fail to capture the intricate cellular interactions and physiological processes that occur within the placental tissue (Kapałczyńska et al., 2018; Kuo et al., 2018). Researchers have employed various 2D culture systems to study placental function. For instance, trophoblast monolayer cultures—both conventional (JEG-3, BeWo, HTR8/SVneo cells) and Transwell systems—have been used to investigate placental barrier properties and drug transport (Rabussier et al., 2023; Olivier et al., 2021). Similarly, BAP-derived trophoblast cultures, cell models that differentiate human pluripotent stem cells into trophoblast-like cells using a cocktail of BMP4, A83-01, and PD173074, also have limitations (Sheridan et al., 2022). These 2D systems are short-lived, yield heterogeneous cell populations, and lack the spatial organization necessary to reflect, authentic placental architecture and function (Cherubini et al., 2021). In contrast, 3D models, particularly organoids, offer a more accurate representation of the placental microenvironment, allowing researchers to investigate developmental processes, disease mechanisms, and drug responses in a physiologically relevant context (Huang et al., 2023; Kapałczyńska et al., 2018; Yang et al., 2022).

This review evaluates current placental organoid models, including primary trophoblast organoids, JEG-3 organoids, and stem cell-derived systems, to determine their strengths and limitations in mimicking human placental development *in-vivo*. By comparing these 3D models with traditional 2D cultures, we highlight their relative physiological relevance and utility in studying key processes such as trophoblast invasion, syncytialization, and placental barrier function. We also discuss how integrating multiple organoid systems with advanced technologies and bioinformatics approaches offers the most comprehensive strategy for modeling the placenta across all developmental stages.

### Placenta and trophoblast cells

The placenta is a unique organ that develops during pregnancy, serving as the critical interface between mother and fetus (Furukawa et al., 2014). This disc-shaped structure, formed from the trophectoderm of the blastocyst upon implantation, plays essential roles in nutrient and gas exchange, hormone production, and fetal protection (Furukawa et al., 2014; Burton and Fowden, 2015). The placenta’s complex structure is composed of chorionic villi, which are vascular projections of fetal tissue surrounded by chorion, extending into the intervillous space where maternal blood pools (Benirschke and Kaufmann, 1992; Griffiths and Campbell, 2014).

At the core of placental function are specialized cells called trophoblasts, which form the epithelial covering of the placenta and are crucial for its development and function (Lawless et al., 2023; Lunghi et al., 2007). There are three main types of trophoblasts in the human placenta: Cytotrophoblasts (CTBs), which are mononucleated cells and act as a stem cell pool, regenerating other trophoblast types and contributing to placental energy production (Lawless et al., 2023). Syncytiotrophoblasts, which are formed by the fusion of cytotrophoblasts, area multinucleated layer that covers the placental surface and is in direct contact with maternal blood, facilitating material exchange between mother and fetus (Griffiths and Campbell, 2014; Lawless et al., 2023). The final type of trophoblasts are extravillous trophoblasts (EVTs) (Menkhorst et al., 2016). These cells invade the maternal uterus, anchoring the placenta and remodeling maternal spiral arteries to ensure optimal perfusion (Lawless et al., 2023; Lunghi et al., 2007). Trophoblasts appear as early as 4 days after fertilization in humans and are essential for embryo implantation and placental development (Menkhorst et al., 2016). Their proper differentiation and function are critical for a healthy pregnancy, as abnormalities in trophoblast development can lead to complications such as preeclampsia and placental insufficiency (Lunghi et al., 2007; Raguema et al., 2020).

Given the essential role of these cells in pregnancy outcomes, there is a growing need for models that accurately recapitulate their behavior *in-vivo*. Recent advancements in cell culture techniques have led to the development of 3D *ex vivo* models known as trophoblast organoids (Raguema et al., 2020; Pascual, 2022) to study these distinct cell types. These organoids mimic the key features of the human placenta, providing researchers with a valuable tool to investigate trophoblast biology and maternal-fetal interactions, and can be made from current trophoblasts and stem cell-derived trophoblasts (Pascual, 2022). In the following sections, this review will explore the recent developments in trophoblast organoid models and their applications in studying placental biology and pregnancy-related disorders.

### Types of 3D organoid models

#### Trophoblast organoids (derived from the placenta)

**FIGURE 2:**
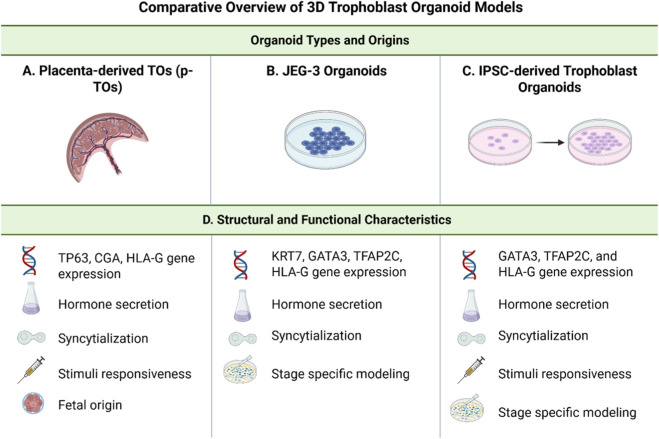
Comparative overview of 3D Trophoblast Organoid models. This figure provides a side-by-side comparison of three trophoblast organoid models commonly used to study human placental development: placenta-derived trophoblast organoids (p-TOs), JEG-3 organoids, and induced pluripotent stem cell (iPSC)-derived trophoblast organoids. Organoid types and origins. **(A)** p-TOs are established from early gestation placental and retain the native trophoblast architecture. **(B)** JEG-3 organoids are derived from the choriocarcinoma JEG-3 cell line and serve as a reproducible, long-term culture system. **(C)** iPSC-derived trophoblast organoids are generated by directed differentiation of pluripotent stem cells and offer developmental modeling across gestational stages. **(D)** Structural and functional characteristics. All models express key trophoblast gene markers, secrete pregnancy-associated hormones (e.g., hCG), and are capable of syncytialization. p-TOs uniquely retain a fetal tissue origin and demonstrate responsiveness to differentiation cues. JEG-3 organoids are limited in structural complexity but support stable, stage-specific modeling. iPSC-derived organoids are stimuli-responsive and can recapitulate multiple stages of trophoblast development, providing a scalable and patient-specific platform for placental research. Created in BioRender (2025). https://BioRender.com/ik6h3uo.

Recent advancements in cell culture techniques have led to the development of *ex vivo* models of trophoblast organoids derived from placental tissues (p-TOs) (Deloria et al., 2021; Haider et al., 2018; Turco et al., 2018). P-TOs are miniature, self-organizing structures that are derived from placental tissue (Figure 2A). They offer a unique opportunity to study the intricacies of placental development and function in a controlled laboratory setting (Barry et al., 2024; Karvas et al., 2022). P-TOs can be generated using various methods, primarily involving the isolation of trophoblast cells from human placental tissues at different gestational stages (Turco et al., 2018; Karvas et al., 2022). One approach utilizes mid-to-late gestation human placental tissue as a source of trophoblast cells, while others have developed techniques for establishing p-TOs from earlier stages, including first-trimester placentas (Karvas et al., 2022; Shannon et al., 2024). Specifically, trophoblasts in early gestation are highly proliferative, invasive, and more plastic, reflecting their critical role in placental development and uterine remodeling during the establishment of pregnancy (Lawless et al., 2023; Knöfler et al., 2019). These intrinsic properties likely enhance their capacity for long-term self-renewal *in-vitro* and structural organization for organoid development (Haider et al., 2018; Zhuang et al., 2023a). In contrast, trophoblasts from late gestation placentas exhibit reduced proliferative potential and a shift towards terminal differentiation, including increased syncytialization and altered signaling pathways, which may limit their ability to expand and self-organize in *in-vitro* culture systems (Higuchi et al., 2019; Sultana et al., 2018). To isolate these cells for study, researchers typically employ enzymatic digestion of placental tissue—often using trypsin, collagenase, or dispase—to dissociate villous cytotrophoblasts from the extracellular matrix. The resulting cell suspension is then subjected to selective culture conditions, including Matrigel-based 3D matrices and defined trophoblast-supporting media supplemented with growth factors such as EGF, FGF4, and heparin. These conditions promote the survival, proliferation, and self-organization of trophoblast progenitors into organoid structures that recapitulate key features of placental architecture and function (Karvas et al., 2022). The benefits of using trophoblasts from the placenta to generate TOs is that they create a more natural mimic of the trophoblasts (Hori et al., 2024).

#### Key characteristics and validation of trophoblast organoids

P-TOs exhibit several key characteristics that make them representative models of placental tissue (Figure 2D). They express trophoblast-specific markers, including CDX2, GATA3, KRT7, and TFAP2C, which are crucial for maintaining trophoblast identity (Yang et al., 2024; Karvas et al., 2022). P-TOs demonstrate functional properties similar to those of the placenta *in-vivo*, such as the secretion of pregnancy-related hormones like human chorionic gonadotropin (hCG) (Yang et al., 2024; Turco et al., 2018). Structurally, p-TOs form complex, villous-like arrangements that closely resemble the architecture of placental villi, including the formation of syncytiotrophoblast-like structures, which are multinucleated layers playing a critical role in placental function (Turco et al., 2018; Sheridan et al., 2021). These organoids also show responsiveness to various stimuli, such as forskolin treatment, which can induce further differentiation and syncytialization, mimicking processes observed in the developing placenta (Zhuang et al., 2023b).

Validation experiments have confirmed the appropriateness of p-TOs as a model system (Yang et al., 2024; Turco et al., 2018; Author Anonymous, 1990; Li et al., 2024). Genetic analysis, including microsatellite analysis and HLA typing, has been used to confirm the fetal origin of p-TOs (Turco et al., 2018). Comparative genomic hybridization (CGH) array analysis has demonstrated long-term culture stability, showing that p-TOs maintain genomic stability over multiple passages (Wessel et al., 2024). Functional assays have assessed the viability and functionality of p-TOs through mitochondrial activity assays and hormone secretion measurements (Turco et al., 2018).

#### Challenges and advancements in trophoblast organoid models

Despite these advances, the p-TO model faces some challenges. One significant difficulty is the variability in cellular orientation within the organoids (Yang et al., 2024). In standard culture conditions, the syncytiotrophoblast (STB) often develops on the inner surfaces of the organoids, which does not accurately reflect the *in-vivo* arrangement of chorionic villi, and they require a complicated isolation process (Hori et al., 2024). To address this limitation, researchers have made significant strides in improving the physiological relevance of TOs (Knöfler et al., 2019; Wessel et al., 2024). Recently, a method has been developed to culture TOs in suspension with gentle agitation, leading to the formation of organoids with the STB on the outer surface (Hori et al., 2024). This arrangement more closely mimics the cellular orientation of chorionic villi *in-vivo* (Hori et al., 2024). Single organoid culture using U-bottomed ultra-low attachment 96-well plates has allowed for more controlled experiments and analysis of organoid formation efficiency (Karvas et al., 2022). Advanced imaging techniques, such as whole-organoid immunostaining followed by 3D confocal microscopy, have been employed to better define the localization of key trophoblast cell populations within the organoids (Turco et al., 2018). Additionally, researchers have developed protocols for inducing EVT differentiation within TOs, further expanding their utility in studying various aspects of placental biology (Wessel et al., 2024).

#### Summary

Although p-TO development can be complex, recent advancements such as advanced imaging techniques using more complex technology have significantly improved the physiological relevance of TOs, making them an increasingly valuable tool for studying human placental development, function, and pathology (Haider et al., 2024; Sato et al., 2021). However, it is essential to note that while TOs offer many advantages, they still represent a simplified model of the complex *in-vivo* placental environment and should be used in conjunction with other research methods for comprehensive studies of placental biology (Karvas et al., 2022).

#### JEG-3 organoids

Placental cell lines have been developed as valuable tools for studying placental biology, trophoblast function, and pregnancy-related disorders (Li et al., 2023; Rothbauer et al., 2017; Weber et al., 2021). These cell lines were created to overcome the limitations of primary trophoblast cultures, which have restricted proliferative capacity and short-term viability (Li et al., 2023). Typically derived from choriocarcinomas, placental cell lines allow for continuous culture and experimentation (Rothbauer et al., 2017; Liu et al., 2023). Among these, JEG-3 cells have emerged as a beneficial model (Figure 2B) (Dietrich et al., 2021). JEG-3 cells are a human choriocarcinoma cell line that has gained prominence in placental research due to several advantages (Dusza et al., 2023; Olivier et al., 2021; Zhuang et al., 2023b). They provide a reliable *in-vivo* placental model for studying trophoblast behavior and placental syncytialization (Figure 2D) (Olivier et al., 2021). It is important to clarify a common misconception regarding 3D culture models derived from trophoblast, specifically the distinction between spheroids and organoids (Olivier et al., 2021). While both represent 3D systems, they differ substantially in biological complexity and developmental potential (Zhuang et al., 2023b; Dietrich et al., 2021).

Spheroids typically arise from forced aggregation methods such as hanging drops or ultra-low attachment plates (Raghavan et al., 2016). These models are valuable for studying certain aspects such as drug responses or cell–cell interactions, but they lack the tissue architecture and self-organization capacity characteristic of organoids (Gunti et al., 2021). In contrast, organoids derived from JEG3 cells are generated within an extracellular matrix scaffold (e.g., Matrigel) under defined culture conditions that support cellular polarization, lumen formation, and multilineage differentiation (Zhuang et al., 2023b; Dietrich et al., 2021). However, it is important to note that evidence for distinct trophoblast lineages in JEG3-derived organoids remains limited (Zhuang et al., 2023b). While these structures express general trophoblast markers (e.g., KRT7, GATA3, TFAP2C), reports of lineage-specific markers such as syncytiotrophoblast genes (CGA, CGB, ERVW-1) or cytotrophoblast stem markers (TP63) are sparse (Zhuang et al., 2023b; Dietrich et al., 2021; Wu et al., 2023). Therefore, the description of multilineage differentiation primarily reflects their structural organization rather than confirmed molecular lineage specification, which requires further validation (Li et al., 2023; Dietrich et al., 2021). However, these features mimic key aspects of placental tissue architecture and function and closely resemble organoids derived from primary trophoblasts, supporting the use of the term “organoid” in this context (Dietrich et al., 2021).

Additionally, primary trophoblast organoids (pTOs) express cytotrophoblast stem-associated markers such as TP63 and lineage-specific genes including CGA/CGB and HLA-G, while iPSC-derived TOs similarly activate trophoblast programs (GATA3, TFAP2C) but often retain low-level pluripotency markers and show less robust EVT differentiation (e.g., reduced HLA-G expression) (Haider et al., 2018; Karvas et al., 2022; Shannon et al., 2024) (Figure 2D). These cells secrete hormones such as hCG and placental lactogen, making them valuable for hormonal regulation studies (Olivier et al., 2021; Hardman et al., 2007). JEG-3 cells are considered closer to human physiology compared to other placental cell lines, making them a standard tool for assessing chemical toxicity in the placenta (Olivier et al., 2021).

#### Key characteristics and validation of JEG-3 organoids

Multiple studies have identified JEG-3 cells as a robust model for recapitulating the human placental barrier, as they exhibit key functional characteristics of both cytotrophoblasts and syncytiotrophoblasts (Hautefort et al., 2022). RNA-seq analyses further support their relevance, revealing that the transcriptional profile of JEG-3 cells closely aligns with that of primary trophoblasts (McConkey et al., 2016). Notably, when cultured in 3D, JEG-3 organoids demonstrate immune barrier properties comparable to the syncytialized trophoblast layer and exhibit a similarly high resistance to viral infection (Blanchon et al., 2002). In long-term culture, JEG-3 cells develop into cellular sheets, due to their morphology, which is advantageous for studying transepithelial transport (Fogh et al., 1977). Additionally, these cells respond to hormonal stimuli, facilitating studies on the regulation of trophoblast calcium transport (Tuan et al., 1991).

#### Challenges and advancements in JEG-3 organoid models

Despite their widespread use, JEG-3 cells have notable limitations that may restrict their utility in placental research (Olivier et al., 2021; Zhuang et al., 2023b; Dietrich et al., 2021; Huang et al., 2024). As a choriocarcinoma-derived cell line, they do not fully recapitulate the behavior of normal placental cells (Zhuang et al., 2023b). Their monocellular nature also prevents them from mimicking the complex multicellular interactions characteristic of the *in-vivo* placental environment (Dietrich et al., 2021). Compared to other trophoblastic cell lines such as BeWo and JAR, JEG-3 cells are often favored for their closer physiological resemblance to primary human trophoblasts (Olivier et al., 2021; Rothbauer et al., 2017; Zhao et al., 2021) making them a valuable model for studying specific aspects of placental pathology. However, to achieve a more comprehensive understanding of placental biology, JEG-3 cells should be complemented with additional models—such as primary trophoblast cultures, 3D organoids, or computational approaches like artificial intelligence (AI) models that better capture the structural and functional complexity of the placenta (Chou and Goldstein, 2025). Emerging AI models can integrate multi-omics datasets, predict cell–cell interactions, and simulate placental development under various physiological and pathological conditions, thereby enhancing model accuracy and guiding experimental design (de Oliveira et al., 2024).

#### Summary

Despite the limitations of JEG-3 cells as a choriocarcinoma-derived line, researchers have made significant strides in developing more advanced placental models that leverage their strengths while addressing key shortcomings. One notable advancement is the generation of JEG-3-derived organoids, which offer greater structural complexity and physiological relevance compared to traditional 2D cultures (Zhuang et al., 2023b; Dietrich et al., 2021). These 3D structures combine the reproducibility and accessibility of JEG-3 cells with the enhanced modeling capabilities of organoids, creating a powerful platform for studying placental biology (Zhuang et al., 2023b). By combining the reproducibility and availability of JEG-3 cells with the structural complexity of organoids, scientists have created a powerful new tool for investigating placental biology (Zhuang et al., 2023b). Although JEG-3 organoids inherit some limitations from their cancerous origin, they effectively complement primary trophoblast organoids (p-TOs) by providing a consistent and scalable model system (Li et al., 2023). Importantly, JEG-3 organoids retain the ability to mimic key aspects of early trophoblast development, including marker expression and differentiation potential, making them a valuable resource for investigating human placentation (Dietrich et al., 2021; McConkey et al., 2016).

#### Stem cell-derived organoids

Given what we know about JEG-3 organoids and p-TOs, researchers have also made significant strides in developing trophoblast organoids derived from pluripotent stem cells (TOs) (Figure 2C) (Shannon et al., 2024). These stem cell-derived organoids offer unique advantages in modeling human placental development and function across various gestational stages (Shannon et al., 2024). TOs can be generated from two main types of pluripotent stem cells: induced pluripotent stem cells (iPSCs) and embryonic stem cells (ESCs) (Castel et al., 2020; Gerami-Naini et al., 2004). IPSCs are reprogrammed from adult somatic cells, providing the advantage of patient-specific modeling, while ESCs, derived from blastocysts, offer a more standardized starting point for trophoblast differentiation (Liang and Zhang, 2013). Both iPSCs and ESCs can be directed towards trophoblast lineages using specific differentiation protocols, such as the introduction of certain WNT inhibitors and the introduction of BMP4, resulting in organoids that mimic key features of the human placenta. However, iPSCs do not require cells from a blastocyst (Liang and Zhang, 2013).

#### Key characteristics and validation of JEG-3 organoids

A key advantage of stem cell-derived TOs is their ability to model various stages of placental development (Yang et al., 2024; Yang et al., 2022). They can mimic the initial stages of trophoblast lineage specification and villous formation, providing insights into implantation and early placental establishment (Shannon et al., 2024). As the organoids mature, they develop more complex villous structures and begin to exhibit functional characteristics of the mid-gestation placenta, such as hormone production and nutrient transport (Shannon et al., 2024). With extended culturing and appropriate stimuli, some stem cell-derived organoids can model aspects of late-stage placental function, including syncytialization and the expression of term-specific markers such as HCG and HLA-G (Yang et al., 2024; Shannon et al., 2024).

The ability to model these different stages within a single system allows for the study of placental changes, offering opportunities to investigate the mechanisms underlying normal and pathological placentation (Figure 2D). TOs complement existing models by providing a renewable source of human trophoblast tissue that can be genetically manipulated and studied (Haider et al., 2024). This approach holds great promise for advancing our understanding of human placental biology and developing new strategies to address pregnancy-related disorders.

#### Advantages of stem- cell derived organoid models

The use of 3D organoid models in placental research offers several key advantages over traditional monolayer cell culture systems. These advantages stem from the organoids’ ability to mimic the complex structure and function of the human placenta in a 3D plane (Cherubini et al., 2021; Dietrich et al., 2021). 3D organoid models provide a higher degree of replication compared to conventional 2D cultures because they can create a more comprehensive model of the cell tissue in question (Ong et al., 2025; Suarez-Martinez et al., 2022). TOs, JEG-3 organoids, and stem cell-derived organoids all create more realistic cellular environments that resemble the physiological conditions of the placenta *in-vivo* (Shannon et al., 2024; Dietrich et al., 2021; McConkey et al., 2016). This replication leads to more reliable, rigorous, and reproducible experimental results (Yang et al., 2024).

In 3D organoid cultures, cells can interact in a manner closer to their natural environment (Haider et al., 2024). This includes complex cell-cell and cell-matrix interactions that play crucial roles in various cellular processes such as proliferation, differentiation, and migration (Haider et al., 2024). These interactions are particularly important in studying trophoblast function and the formation of syncytiotrophoblast-like structures because they can help researchers understand drug-drug interactions and the effects of diseases on these different placental cell types (Yang et al., 2024; Hamilton et al., 2023). The 3D organoid models are especially valuable in studying the complex architecture of the human placenta, including the formation of villous structures and the differentiation of EVTs (Hamilton et al., 2023).

#### Challenges in stem- cell derived organoid models

Stem-cell-derived placental organoids offer a promising platform to model early human trophoblast development, but they are limited by several technical and biological challenges. Differentiation protocols often yield heterogeneous cell populations, with variable efficiency in generating CTB, STB, and EVT subtypes depending on cell line, media conditions, and induction strategy (Turco et al., 2018; Hori et al., 2024; Horii et al., 2019). While these models offer greater accessibility and patient-specific potential than primary cultures, they frequently lack the structural organization and dynamic temporal cues necessary to recapitulate the maternal–fetal interface (Hori et al., 2024; Zhou et al., 2025). For instance, STB often forms in non-physiological locations within organoids, and maternal cell types like decidual stromal or immune cells are absent, limiting the model’s utility in studying implantation or immune tolerance (Yang et al., 2024). Moreover, these organoids may display epigenetic aberrations such as abnormal imprinting, and altered DNA methylation compared to primary trophoblasts, raising concerns about developmental fidelity and long-term stability (Dong et al., 2020).

Reproducibility, scalability, and ethical concerns further complicate the widespread use of stem-cell-derived placental models (Shannon et al., 2024). Protocols often show batch-to-batch variability, and inconsistencies in trophoblast marker usage across studies make it difficult to establish standardized benchmarks (Schäffers et al., 2025). Additionally, extended culture timelines and technical complexity limit scalability (Karvas et al., 2022). Although iPSCs mitigate some ethical concerns associated with embryonic stem cells, regulatory hurdles remain, particularly for patient-specific or genome-edited lines intended for therapeutic use (Hockemeyer and Jaenisch, 2016; Madrid et al., 2024). These limitations highlight the need to combine stem-cell-derived models with complementary systems to more fully capture the structural, molecular, and functional complexity of human placental development.

#### Summary

Compared to traditional cell cultures, 3D organoid models often support longer-term culture and functionality of placental cells (Cherubini et al., 2021). This prolonged culture duration is especially advantageous for investigating time-dependent processes such as placental development and hormone production, as well as for assessing disease progression and responses to environmental factors or therapeutic interventions that require extended observation to capture their effects fully (Yang et al., 2024). 3D organoid models also demonstrate improved functionality similar to *in-vivo* placental tissue (Yang et al., 2024). This is evident in their ability to mimic gene expression profiles that more closely match those of primary tissue, and the formation of barrier structures similar to the placental barrier (Yang et al., 2024; Haider et al., 2018). These models also provide a unique platform for studying placental resistance to microbial infections (Yang et al., 2022; McConkey et al., 2016). The model has shown resistance to viral and parasitic infections like *Toxoplasma gondii* that more closely resembles the behavior of *in-vivo* syncytiotrophoblast, making them valuable tools for investigating placental host-pathogen interactions (McConkey et al., 2016).

### Comparison to other models

Placental biology research has made significant strides through the use of established cell lines and primary cell cultures, which have enabled the development of *in-vivo* models to investigate the complex maternal–fetal interface and key processes in placental development (Liu et al., 2023; Aengenheister et al., 2018). These systems have provided critical insights into trophoblast differentiation, hormone secretion, and barrier function, forming the bedrock of experimental placental biology. Primary trophoblast cells offer high physiological relevance but are often limited by short lifespans, donor variability, and technical challenges associated with isolation (Piwocka et al., 2024; Rosario et al., 2021). In contrast, cell lines such as BeWo, JEG-3, and HTR-8/SVneo are more accessible and reproducible but frequently lack the full spectrum of functional characteristics seen in primary placental cells (Li et al., 2023; Abou-Kheir et al., 2017). This may reflect fundamental biological differences: HTR8/SVneo cells are immortalized first-trimester extravillous trophoblasts transformed with simian virus 40 large T antigen (SV40) (Graham et al., 1993), which confers unlimited growth potential but also alters key signaling pathways governing differentiation and cell polarity (Weber et al., 2013). As a result, these cells exhibit a strong invasive phenotype but limited plasticity and differentiation potential, which likely constrains their ability to self-organize in 3D (Weber et al., 2013). By contrast, JEG3 cells retain a degree of proliferative capacity and architectural organization that, under appropriate culture conditions, can give rise to organoid-like structures (Dietrich et al., 2021).

To address these limitations, 3D organoid cultures have emerged as a transformative platform by better replicating the architecture and cellular diversity of the placenta (Barry et al., 2024; Haider et al., 2024; Dietrich et al., 2021). Organoids offer improved spatial organization, prolonged viability, and enhanced physiological function compared to traditional monolayer cultures. However, they are not without drawbacks. Limitations such as differentiation efficiency, structural and temporal fidelity, epigenetic stability, reproducibility, scalability, and regulatory hurdles suggest that there is a need to integrate these models with complementary systems (Hori et al., 2024; Zhou et al., 2025).

Additional models can be more advanced technologies, and have been developed to further enhance the modeling of placental biology. For example, the Membrik System is a microfluidic-based co-culture platform that allows researchers to compartmentalize maternal and fetal cell types while simulating mechanical forces such as flow and pressure (Boos et al., 2021). This enables dynamic modeling of nutrient transport, immune cell migration, and vascular remodeling with higher physiological fidelity. In parallel, AI driven approaches are being used to integrate multi-omics data, predict cell–cell interactions, and simulate pathological states such as preeclampsia or intrauterine growth restriction (Chou and Goldstein, 2025; Wang H. et al., 2024). These AI models allow for the rapid analysis of complex datasets and can generate predictive insights that are difficult to derive from wet-lab experiments alone (Chou and Goldstein, 2025).

While these methods have significantly advanced the field and demonstrated predictive capabilities for various pathologies, it is essential to acknowledge that these current models still have inherent limitations (Kapałczyńska et al., 2018). For example, most AI models cannot fully replicate the dynamic physiological conditions of pregnancy or account for all the cell types present in the placental barrier (Zhou et al., 2023). Additionally, factors like oxygen tension, hormonal influences, and immune system interactions are challenging to replicate *in-vivo* (Cherubini et al., 2021).

### Applications of placental research

Placental research has made significant strides, providing insights into trophoblast biology, placental barrier function, and pregnancy-related pathologies (Cherubini et al., 2021). Applications of placental research, such as understanding pregnancy complications, placental drug transport studies, and infectious disease, have the potential to open the door for more research on the placenta (Mao and Chen, 2021; Megli and Coyne, 2022). Trophoblast invasion and differentiation are critical processes in placental development and successful pregnancy (Dietrich et al., 2021). Current research has been used to understand the differentiation of trophoblast cells and their complex molecular pathways (Schäffers et al., 2022). Recent research has also identified key transcription factors and signaling molecules that govern trophoblast lineage specification and differentiation (Yang et al., 2022; Okae et al., 2018). For example, the Wnt signaling pathway has been shown to play a crucial role in regulating trophoblast proliferation and invasion (Aengenheister et al., 2018; Okae et al., 2018).

Other applications involve understanding the pathways used to establish EVTs to ensure proper placentation without excessive invasion, which can lead to limited understanding (Hamilton et al., 2023). Studies have revealed that this process is controlled by a network of specific growth factors, cytokines, and adhesion molecules (Yang et al., 2024; Hamilton et al., 2023). The balance between pro-invasive and anti-invasive factors is crucial for maintaining appropriate trophoblast invasion, which could result in intrauterine growth restriction or early pregnancy loss (Yang et al., 2024).

Syncytialization, the fusion of cytotrophoblasts to form the multinucleated syncytiotrophoblast, is essential for placental function (Keenen et al., 2024). Syncytialization occurs on the outermost surface of the placental villi and is responsible for hormone production, immune protection, and trophoblast turnover (Pötgens et al., 2002). Recent research has uncovered the molecular mechanisms involved in this process, including the role of proteins and the regulation of the cell cycle (Yang et al., 2024). Understanding these mechanisms is crucial for comprehending placental development and function.

Other applications, such as placental barrier models, have been instrumental in studying the mechanisms of nutrient and gas exchange across the placenta (Turco et al., 2018). These studies have provided insights into how the placenta regulates fetal growth and development by controlling the transfer of essential substances (Turco et al., 2018). These models have also facilitated active research in elucidating the placental origins of preeclampsia and other pathologies such as gestational diabetes and placental infections (Karvas et al., 2022; Hori et al., 2024; Zhu et al., 2023).

### Bioinformatics approaches

Computational analysis of transcriptomic data has become an essential tool for characterizing placental organoids and comparing them to natural placental tissue (Cherubini et al., 2021). These bioinformatics approaches allow researchers to assess the fidelity of organoid models and identify key molecular features of placental development (Turco et al., 2018; Karvas et al., 2022; Shannon et al., 2024; Wang M. et al., 2024).

RNA sequencing (RNA-seq) has emerged as the primary method for transcriptome-wide gene expression profiling (Kukurba and Montgomery, 2015). Key steps involve quality control of raw sequencing data, mapping reads to a reference genome, and normalizing expression values across samples (Kukurba and Montgomery, 2015).

Comparing the transcriptomes of placental organoids to primary placental tissue is crucial for validating organoid models. Current research groups utilize bioinformatics approaches such as Pearson and Spearman correlation coefficients to measure global gene expression, principal component analysis (PCA) to visualize overall transcriptome similarity, and Gene Set Enrichment Analysis (GSEA) to compare pathway activation (Yang et al., 2024; Yang et al., 2022). While TOs showed high overall similarity to natural placenta, some differences in gene expression patterns were observed, particularly related to tissue maturation and *in-vivo* microenvironmental cues (Yang et al., 2024).

Current research has analyzed temporal gene expression patterns across early, mid, and late stages of placental development using both primary tissue and organoid models (Yang et al., 2024; Turco et al., 2018). They have identified early, mid, and late stage-specific markers through differential expression analysis (Yang et al., 2024; Turco et al., 2018). Clustering analysis revealed distinct transcriptional programs activated at each developmental stage, with early stages characterized by stem cell maintenance and proliferation genes, mid stages by differentiation and hormone production genes, and late stages by genes involved in nutrient transport and immune modulation (Paquette et al., 2018).

Single-cell RNA-seq (scRNA-seq) has revolutionized the study of cellular heterogeneity in placental organoids (Zhuang et al., 2023a). This technique allows researchers to identify distinct cell populations and subtypes, reconstruct developmental trajectories, and uncover rare cell types (Zhuang et al., 2023a). ScRNA-seq has revealed previously unappreciated diversity in trophoblast populations and enabled detailed mapping of differentiation pathways in placental organoids (Zhuang et al., 2023a). Building on this, spatial transcriptomics offers a complementary approach by preserving the spatial context of gene expression, while enabling researchers to localize transcriptional activity within distinct regions of TOs.

Emerging spatial transcriptomics methods combine gene expression profiling with spatial information, allowing researchers to map transcriptional patterns to specific regions within placental organoids (Robles-Remacho et al., 2023). These techniques are particularly valuable for studying the spatial organization of different trophoblast subtypes and modeling the complex 3D architecture of the placenta because of the impact they have on placental research (Arutyunyan et al., 2023).

By integrating these multi-level bioinformatics approaches, researchers can comprehensively understand gene expression dynamics in placental organoids and how they relate to *in-vivo* placental development. However, these findings can lead to more challenges in the field.

### Challenges and limitations of TOs

Research in placental biology faces several significant challenges and limitations. Standardization issues are a primary concern (Turco et al., 2018; Wang M. et al., 2024). There is considerable variability in organoid generation protocols, leading to reproducibility challenges across different laboratories (Liu et al., 2023). This variability can result in inconsistencies in experimental outcomes, making it difficult to compare results from different studies (Liu et al., 2023). Consequently, there is a need for quality control measures to ensure consistent and reliable results in placental research.

In addition to standardization challenges, ethical considerations play a crucial role in organoid research (de Jongh et al., 2022). The use of human tissue samples raises critical ethical questions regarding the sourcing of these materials, especially in early gestation (de Jongh et al., 2022). Researchers must navigate the complexities of informed consent processes to ensure that donors are fully aware of how their tissues will be used in research (de Jongh et al., 2022). Furthermore, there is an ongoing need to develop and refine ethical guidelines tailored explicitly to organoid research to address these complex issues and protect the rights of tissue donors (de Jongh et al., 2022). These limitations not only affect p-TOs, but also other models.

### Future directions

The field of organoid research holds promising future directions that could significantly advance our understanding of human biology and disease. One exciting avenue of development is the integration with other organ systems (Elzinga et al., 2023; Mosavati et al., 2020). Multi-organ-on-a-chip models offer the potential to more accurately model systemic maternal-fetal interactions, providing a more comprehensive understanding of placental function in the context of the entire body (Mosavati et al., 2020). Such integrated models could enhance our ability to study complex physiological processes and their implications for health and disease.

The field of organoid research offers exciting future directions that promise to revolutionize our understanding of human biology and disease, especially in the context of pregnancy and placental biology. The integration of placental organoids with other organ systems, creating interconnected microenvironments that better mimic *in-vivo* physiology (Wang et al., 2018). For example, multi-organ physiological systems, also known as body-on-a-chip platforms, enable the simultaneous culture of multiple tissue types under dynamic flow conditions, mimicking systemic nutrient delivery, hormonal feedback, and immune signaling (Wang H. et al., 2024; Wang et al., 2018). Specifically, recent studies have employed organ-on-chip technologies to engineer placental organoids within vascularized niches, integrating trophoblast layers with fetal endothelial barriers under controlled flow (Deng et al., 2022; Lermant et al., 2024; Wang et al., 2025).

Notably, Wang et al., 2024 developed a human placental organoid microphysiological system within a vascular niche, providing a dynamic and perfused environment that supports sustained tissue viability and functional trophoblast differentiation (Wang et al., 2025). This engineered platform successfully models viral infection and immune responses, enabling real-time analysis of pathogen transmission across the placental barrier (Wang et al., 2025). By integrating organoid biology with vascular flow and immune components, such systems bridge the gap between static 3D cultures and *in-vivo* physiology (Wang et al., 2025). Moving forward, these organoid-on-chip approaches hold immense potential for investigating maternal-fetal pathogen interactions, drug transfer, and placental barrier function under controlled and physiologically relevant conditions, thereby advancing translational research in reproductive and developmental biology (Wang et al., 2025). This model, and other complex models supports improved differentiation, innate immune responses, and long-term viability compared to static culture systems (Deng et al., 2022; Lermant et al., 2024; Wang et al., 2025). Collectively, these approaches streamline the study of maternal–fetal interactions, enabling advanced modeling of pathologies such as preeclampsia, gestational diabetes, and drug exposure.

Furthermore, personalized medicine applications represent another Frontier in organoid research (Schäffers et al., 2022). Patient-specific organoids could revolutionize drug screening and toxicity testing, allowing for more tailored and effective treatments based on individual patient profiles and epigenetic modifications (Schäffers et al., 2022). These advanced organoid models also hold promise for developing predictive models for pregnancy complications, potentially improving maternal and fetal health outcomes (Schäffers et al., 2022). By leveraging the unique capabilities of organoids, researchers can move toward more personalized healthcare approaches that address patients’ specific needs.

## Conclusion

3D organoid models have revolutionized placental research, offering significant advantages over traditional methods in terms of physiological relevance and functional similarity to human placental tissue (Kapałczyńska et al., 2018; Fry et al., 2019; Kreuder et al., 2020). The integration of multiple approaches, including trophoblast organoids, JEG-3 organoids, and stem cell-derived organoids, coupled with advanced technologies and bioinformatics analyses, currently provides the most comprehensive strategy for mimicking placental development across all stages *in-vivo*. Bioinformatics approaches, particularly transcriptomic comparisons and gene expression profiling, have been crucial in validating these models and identifying stage-specific markers (Karvas et al., 2022; Keenen et al., 2024; Paquette et al., 2018). However, challenges remain, including standardization issues and ethical considerations (Turco et al., 2018). While no single method perfectly replicates all stages of placental development, the combination of various models, supported by advanced technologies and computational analyses, offers the most effective approach to studying placental biology *in-vivo*. Continued refinement and integration of these methods will be essential for further advancements in the field.
